# Evaluation of Paraoxonase Activity in Children With Nephrotic Syndrome

**DOI:** 10.5812/numonthly.12606

**Published:** 2013-11-13

**Authors:** Mohammad Hashemi, Simin Sadeghi-Bojd, Mohsen Raeisi, Abdolkarim Moazeni-Roodi

**Affiliations:** 1 Cellular and Molecular Research Center, Zahedan University of Medical Sciences, Zahedan, IR Iran; 2 Department of Clinical Biochemistry, School of Medicine, Zahedan University of Medical Sciences, Zahedan, IR Iran; 3 Research Center for Children and Adolescents Health, Zahedan University of Medical Sciences, Zahedan, IR Iran; 4 Department of Pediaterics, School of Medicine, Zahedan University of Medical Sciences, Zahedan, IR Iran

**Keywords:** Nephrotic Syndrome, Aryldialkylphosphatase, Antioxidants

## Abstract

**Background:**

It has been proposed that reactive oxygen species (ROS) is involved in the pathogenesis of various diseases. Paraoxonase, a high-density lipoprotein associated enzyme, prevents low-density lipoproteins from oxidation.

**Objectives:**

The aim of the present study was to investigate the serum activities of paraoxonase-1 (PON-1), and aryleterase (ARE) as well as total antioxidant capacity (TAC) in children with nephrotic syndrome in acute and remission phase.

**Patients and Methods:**

The study consisted of 20 patients in acute and remission phases and 23 healthy controls. PON-1 and ARE activities were determined spectrophotometrically using paraoxone and phenyacetate as substrate, respectively. TAC was measured using ferric reducing ability of plasma (FRAP).

**Results:**

The levels of PON, ARE, and TAC were significantly lower in acute phase of nephrotic syndrome compared with the remission phase. The levels of PON, ARE and TAC increased in remission phase.

**Conclusions:**

Our results revealed that the determination of paraoxonase activity might be a biomarker for responses to nephrotic syndrome treatment, which needs to be fully clarified.

## 1. Background

Nephrotic syndrome, which is more common in children than adults, is mainly a pediatric disorder. It is characterized by heavy proteinuria, hyperlipidemia, hypoalbuminemia and peripheral edema (1). Idiopathic nephrotic syndrome (INS) is one of the most common renal problems in children encountered in day-to-day nephrology practices. Children with INS can be classified into well-defined categories based on the responses to the standard prednisolone therapy (2). Clinically, the best prognostic indicator is the positive or negative responses to the steroid therapy (3), however, it is difficult to predict the steroid responsiveness or steroid resistance (4).

Reactive oxygen species (ROS) are involved in the etiopathogenesis of nephrotic syndrome (NS). It has been proposed that ROS stimulate lipid peroxidation, which is resulted in cell injury; disruption of structural integrity of tubular epithelial cells, enhances glomerular permeability to proteins and changes glomerular hemodynamics (5-7). The term MCD has become synonymous with steroid sensitive idiopathic syndrome, although renal biopsy is usually not used in patients who respond to steroid therapy (8).

Underlying abnormality in nephrotic syndrome is an increase in permeability of the glomerular capillary wall. The cause of the increased permeability is not well understood (1). It is possible that immunological and genetic mechanisms may have roles in this increasing (9). But some recent studies showed that oxygen free radicals may cause glomerular injuries (10), so oxidative stresses, an imbalance between the production of reactive oxygen substances and the antioxidant defensive mechanism, contributes to an enhanced permeability of the glomerular capillary wall (11, 12).

Cellular defense mechanisms against ROS including enzymatic systems such as superoxide dismutase (SOD), catalase, glutathione peroxidase (GPx) and non-enzymatic antioxidant defense system containing albumin, reduced glutathione, uric acid, vitamin C, vitamin E, carotenoids, selenium and zinc. Total antioxidant capacity (TAC) of the system is the sum of endogenous and food-derived antioxidants (13). Antioxidants prevent the production of reactive oxygen substances, so they can play a major role in decreasing the injury in nephrotic syndrome. Albumin is the major and most predominant plasma antioxidant which decreases during active phase of nephrotic syndrome and may be related to nephrotic syndrome (11).

Paraoxonase 1 (PON1) is mainly synthesized in the liver and bind entirely to plasma HDL. Though, its physiological function has not been entirely elucidated, it has been proposed that PON1 hydrolyses lipid peroxides and play a key role in antioxidant system (12). Decreased paraoxonase level can decrease the antioxidant activity of high-density lipoprotein and induce glomerulosclerosis (14). Few studies have investigated that paraoxonase activity and antioxidant status in nephrotic syndrome (14-16).

## 2. Objectives

The present study was aimed to evaluate the oxidant/antioxidant status by measuring paraoxonase and aryl esterase activities as well as total antioxidant capacity in steroid sensitive nephrotic syndrome (before and after treatment) and compares it with healthy control individuals.

## 3. Patients and Methods

### 3.1. Subjects

This prospective study was carried out from February 2009 to August 2010 on patients with nephrotic syndrome in pediatric department of Ali-ebneh Abitaleb Hospital, Zahedan, Iran. Patient who had acute infection, chronic renal failure disease, taking medication during the week before admission, progressed to a steroid dependent course or did not response to a standard regimen of steroid were excluded from this study. Finally 20 children with acute new onset idiopathic steroid sensitive nephrotic syndrome were known as group I. Group II consisted of the same children in the remission phase (urine trace or negative for protein for 3 consecutive days while receiving prednisolone as alternative daily dose). Twenty-three healthy children formed the third group. None of the patients and control groups received any form of antioxidant medications. None of children had microscopic hematuria, azotemia or hypertension. Kidney biopsy was not done. Ethics committee of Zahedan University of Medical Sciences approved the study protocol.

Blood samples were obtained at morning after overnight fasting and instantly separation of serum and plasma were done. The samples were stored at -80 ºC until analysis. Biochemical analysis such as blood urea nitrogen (BUN), creatinine, total cholesterol, triglycerides, HDL- cholesterol, total protein and albumin were carried out by auto-analyzer using commercially available kits.

### 3.2. Paraoxonase and Arylesterase Activities Assay

Determination of paraoxonase and arylesterase activitities was done using paraoxone (diethyl-p-nitrophenyl phosphate) and phenyl acetate as the substrates, respectively, as previously described (17-19). 

### 3.3. Total Antioxidant Capacity (TAC) Assay

TAC of plasma was determined using FRAP (Ferric reducing ability of plasma) assay (20).

### 3.4. Statistical Analysis

The data were expressed as mean ± standard deviation (SD). Statistical analysis was performed using SPSS 18 software by independent sample t-test and paired sample t-test. A P value less than 0.05 was considered statistically significant.

## 4. Results

Demographic and biochemical parameters of nephrotic syndrome in acute and remission phases, and normal subjects were shown in Table 1. There were no significant differences between the groups with regards to the gender (P = 0.225) and age (P = 0.759). The levels of serum total protein and albumin were significantly lower, while the concentration of cholesterol and triglyceride were higher in acute phase of nephrotic syndrome than normal subjects. No significant difference was observed in remission phase and control groups regarding BUN, total protein and serum albumin (P > 0.05).

**Table 1.** Demographic and Biochemical Parameters of Nephrotic Syndrome in Acute Phase, Remission Phase and Normal Subjects

Parameter	Group I (Acute Phase)	Group II (Remission Phase)	Group III (Control)	P Value
**Age, Mean (SD), y**	4.93 (2.16)	-	5.13 (2.19)	0.759
**gender, M/F**	10/10	-	16/7	0.225
**BUN, Mean (SD), mg/dL**	10.55 (3.13)	14.31 (5.81)	14.63 (4.92)	0.034^a^, 0.003^b^, 0.849^c^
**Serum creatinine, Mean (SD), mg/dL**	0.5 (0.16)	0.7 (0.19)	0.58 (0.14)	0.008^a^, 0.113^b^, 0.025^c^
**Total protein, Mean (SD), mg/dL**	4.68 (1.02)	6.59 (0.88)	6.69 (0.8)	< 0.0001^a^, < 0.0001^b^, 0.699^c^
**Serum albumin, Mean (SD), mg/dL**	2.32 (0.49)	4.33 (0.97)	4.67 (0.55)	< 0.0001^a^, < 0.0001^b^, 0.156^c^
**Cholesterol, Mean (SD), mg/dL**	453.68 (102.96)	192.63 (78.07)	147 (31.03)	< 0.0001^a^, < 0.0001^b^, 0.025^c^
**Triglyceride, Mean (SD), mg/dL**	367.84 (206.37)	165.15 (154.73)	92 (52.71)	0.006^a^, < 0.0001^b^, 0.042^c^
**HDL-c, Mean (SD), mg/dL**	51.15 (17.75)	56.09 (14.6)	53 (19.78)	0.164^a^, 0.699^b^, 0.619^c^

^a^Groups I vs. II.

^b^Groups I vs. III.

^c^Groups II vs. III.

As shown in Table 2, the levels of PON, salt-stimulated PON, ARE, and TAC were significantly lower in acute phase of nephrotic syndrome than remission phase (P < 0.05). Although, no significant differences were found between remission phase and normal subjects regarding the PON, salt-stimulated PON, the levels of ARE and TAC were significantly higher in remission compared to the normal subjects. 

**Table 2.** Paraoxonase-1 (PON-1), Salt-stimulated PON-1 (PONS), Aryl Esterase (ARE) Activities and Total Antioxidant Capacity (TAC) in the Active and Remission Phases of Nephrotic Syndrome and in Control Subjects

	Group I (Acute Phase)	Group II (Remission Phase)	Group III (Control)	P Value
**PON-1, Mean (SD), U/L**	73.78 (48.66)	115.74 (59.25)	120.79 (79.27)	0.005^a^, 0.026^b^, 0.816^c^
**PONS, Mean (SD), U/L**	171.42 (111.99)	249.47 (136.89)	214.51 (150.53)	0.025^a^, 0.299^b^, 0.432^c^
**ARE, Mean (SD), kU/L**	77.75 (27.82)	119.59 (27.49)	68.51 (25.92)	0.0001^a^, 0.206^b^, < 0.0001^c^
**TAC, Mean (SD), μmol/L**	874.61 (201.8)	1444.59 (249.31)	1001.84 (218.41)	< 0.0001^a^, 0.050^b^, < 0.0001^c^

^a^Groups I vs. II.

^b^Groups I vs. III.

^c^ Groups II vs. III.

## 5. Discussion

In the present study, significant decreases in serum PON1 and TAC levels were observed in children with acute nephrotic syndrome compared with controls. We also found that at remission phase, the serum paraoxonase level increased and reached to the normal level. In agreement with our findings, Soyoral et al. (16) have found that serum basal and salt-stimulated paraoxonase activities, arylesterase activity and total thiol (SH) levels were significantly lower in patients with NS than in controls. Their findings suggested that low level of PON1 activity in adult patients with NS may be related to atherosclerosis due to oxidant-antioxidant imbalance. Ece et al. (14) have found that patients in the active phase of NS had shown significantly lower PON level and fewer total antioxidant response (TAR), higher oxidative stress index (OSI) and total peroxide values than those in full remission. They found no differences in PON, TAR, or OSI values of relapsing or new-onset NS group and NS with remission plus steroid use.

Kniazewska et al. (15) have investigated TAC, PON-1, α-tocopherols, ascorbic acid in patients who had been treated 4-15 years ago and healthy subjects. They found no statistically significant differences in PON-1 activity, α-tocopherol levels and the sum of β- and γ-tocopherols and TAC between groups.

A variety of PON1 gene polymorphisms have been recognized (21-23). It has been well documented that two common coding region of PON1 polymorphisms (L55M and Q192R), leading to a change of both level and activity of the enzymes (24, 25). Biyikli et al. (26) have found an association between L55M and Q192R polymorphisms of PON1 and focal segmental glomerulonephritis (FSGN). An association between PON1 L55M polymorphism and FSGN was reported by Frishberg et al. (27). It has been suggested that Q192R polymorphism is a risk factor for developing membranoproliferative glomerulonephritis (MPGN) and may be associated with poor prognosis of the disease (28).

Paraoxonase is a serum enzyme binding to high-density lipoprotein (HDL), and has a major role in preventing LDL oxidative modification by hydrolyzing lipid peroxides. An increase of oxidative stress and decrease of PON1 activity were reported in children with chronic renal failure (29).

Some studies expressed that glomerular injury in rats (that is like minimal change disease in human) can be caused by oxidants (5). Antioxidants may play an important preventive role in nephrotic syndrome and its progression, by decreasing the free oxygen radicals (5). Albumin is the most important plasma antioxidant but it is not a chain breaking antioxidant (12, 30). Hyperlipidemia induces the phenotypic changes in microcirculation which are consisted of oxidative stresses leading to pathophysiologic features such as platelet activation, lipid peroxidation and generation of radicals (31). Consequently, hyperlipidemia that increases the lipid oxidation reactions and decreases the antioxidant status, may lead to glomerulosclerosis and progression of glomerular damage in nephrotic syndrome.

In conclusion, the current study on Iranian children showed higher oxidative stress and lower antioxidant activity during acute phase of idiopathic steroid-sensitive nephrotic syndrome. Oxidant-antioxidant imbalance, which is observed during acute phase of nephrotic syndrome, resolved at remission phase. Our results suggest that determination of PON activity may be a marker of an effective treatment of nephrotic syndrome.
